# Shifting the paradigm: physician-authorized, student-led efforts to provide harm reduction services amidst legislative opposition

**DOI:** 10.1186/s13011-021-00362-1

**Published:** 2021-03-24

**Authors:** Timothy P. McMullen, Mahan Naeim, Carol Newark, Haden Oliphant, Jeffrey Suchard, Faried Banimahd

**Affiliations:** 1 University of California, Irvine School of Medicine, Irvine, CA USA; 2 University of California, Irvine Department of Criminology, Law and Society, Irvine, CA USA; 3 American Addiction Institute of Mind and Medicine, Santa Ana, CA USA; 4 Department of Emergency Medicine, University of California, Irvine School of Medicine, Orange, CA USA

**Keywords:** Harm reduction, Syringe services, Syringe exchange, Needle exchange, Injection drug use, Community outreach, HIV, Advocacy, Stigma, Legal obstacles

## Abstract

**Background:**

For over 30 years, syringe services programs (SSPs) have served as an efficacious intervention for the prevention of HIV and Hepatitis C transmission among persons who use drugs. Despite a strong body of evidence for the effectiveness of SSPs as a preventative public health measure, numerous local and state governments in the United States continue to resist the establishment of new SSPs and aggressively pursue the closure of those already in operation.

**Commentary:**

In Orange County, California, local officials have repeatedly mobilized in opposition of the establishment of syringe access – thereby hindering access to healthcare for thousands of predominantly unhoused individuals. The county was previously served by the Orange County Needle Exchange Program from 2016 until 2018 when a civil suit brought by the Orange County Board of Supervisors resulted in the closure of the program. For more than 2 years, persons who inject drugs in Orange County lacked reliable access to clean syringes, placing them at increased risk for contracting HIV and Hepatitis C. Here, we comment on the ongoing effort to restore syringe access in Orange County. This collaborative physician-directed endeavor has brought together students and community volunteers to provide vital harm reduction services to a remarkably underserved population. Since the reestablishment of syringe access in Orange County by the Harm Reduction Institute, new legal barriers have arisen including the passage of new municipal legislation banning the operation of syringe exchanges. We are well-equipped to overcome these obstacles. This work serves as an affirmation of assertions made by previous authors regarding the unique qualifications of medical & graduate students as effective harm reductionists.

**Conclusion:**

Harm reduction services are vital to the health and well-being of people who use drugs. The provision of these services should not be impeded by legislative interference by municipal, county, or state governments.

## Background

Drug overdoses caused 67,367 deaths in the United States in 2018 according to the Centers for Disease Control and Prevention, and opioid overdoses accounted for 46,802 (69.5%) of those deaths [1]. In California, 2410 (45%) of overdose deaths in 2018 were attributable to opioids [2]. The number of Americans at risk for opioid-related morbidity and mortality is significant. The U.S. Department of Health and Human Services estimates that in 2017, 10.5 million Americans misused opioid pain medication and approximately 886,000 Americans used heroin [3]. In the same year, an additional 1.6 million Americans reported using methamphetamine [4]. Intravenous and intramuscular injection drug use carries additional risks beyond that of accidental overdose. Persons who use drugs (PWUD) such as heroin and methamphetamine are at significant higher risk for contracting HIV and Hepatitis C virus (HCV) via reuse and sharing of syringes and needles [5]. An estimated 14.2% of existing U.S. HIV cases and 13.4% of existing California HIV cases were attributable to injection drug use in 2017 [2]. In the same year, an estimated 72.6% of new U.S. HCV cases were associated with injection drug use among patients with reported risk factors [6].

Syringe services programs (SSPs) constitute a well-established, efficacious, harm reduction-based intervention to prevent the transmission of HIV and HCV in populations of PWUDs without increasing the incidence of injection drug use or exacerbating the burden of syringe litter while concurrently facilitating entry into treatment [7–12]. Unfortunately, the implementation of SSPs has been hindered and outright obstructed in many instances due largely to the stigma associated with the use of injection drugs and to a lack of urgency with respect to the population health of low-resource communities. In many cases, local and state officials have resisted the establishment of new SSPs and have worked aggressively to close existing SSPs, driven by incorrect notions about SSPs and their participants. These actions have caused direct harm to PWUDs in communities across the United States. The 2015 HIV outbreak in Scott County, Indiana represents perhaps the best-known example of this phenomenon, particularly since some authors have suggested that the outbreak could have been largely avoided if state officials had acted more quickly to establish an SSP [13–15].

## Legal Basis & Obstacles

The California legislature passed Assembly Bill 136 in 2000 which authorized the establishment of SSPs by municipal and county governments [16]. Bolstered by subsequent legislation regarding the establishment of physician-authorized syringe access, SSPs have proliferated statewide over the last 20 years. Unfortunately, organized opposition to syringe access has blocked the establishment of SSPs in many California counties, mirroring trends elsewhere in the United States [17, 18]. In order to combat local opposition to SSPs, California passed Assembly Bill 604 in 2011, which permits the California Department of Public Health (CDPH) to authorize SSPs in any location where the conditions exist for the rapid spread of HIV, HCV, or any other infectious disease spread through the sharing of used syringes.

In December 2015, the Orange County Needle Exchange Program (OCNEP) became the first SSP to be authorized by the CDPH without express authorization from its city or county government. Despite its position as the third-most populous county in California and the sixth-most populous county in the United States, Orange County had long been without syringe access. The historical absence of services can be attributed to the rejection of syringe access as an evidence-based medical intervention by municipal and county government officials, despite its demonstrated efficacy and a substantial need for these services. According to the Orange County Health Care Agency (OCHCA), lifetime prevalence of heroin use among adults in Orange County has been estimated at 33,000, or approximately 1% of the total population. In addition, a reported 6369 persons living with HIV (PLWH) reside in Orange County, 399 of whom use injection drugs [19, 20].

OCNEP operated in the Santa Ana Civic Center from 2016 until 2018, when the Santa Ana City Council passed an ordinance requiring a permit to provide services in the Civic Center (Section 10–554(a), Santa Ana Municipal Code). OCNEP applied for a permit to continue to provide services but was denied in January 2018. Because CDPH regulations require state-authorized SSPs to comply with local ordinances, OCNEP was forced to close due to the permit denial. In an effort to reopen, OCNEP received CDPH authorization to implement a mobile SSP in four cities across Orange County. Immediately upon receiving its second authorization, Orange County and the cities of Anaheim, Orange, Costa Mesa, and Newport Beach filed a lawsuit against OCNEP and CDPH. Orange County and its co-plaintiffs ultimately won this lawsuit when a judge determined that CDPH had authorized OCNEP without completing an environmental impact report – in violation of the California Environmental Quality Act of 1970. In addition, these four cities passed ordinances making it illegal to operate a CDPH-authorized SSP within the city limits. To date, none of these ordinances have faced legal challenges.

The shuttering of OCNEP left thousands of Orange County residents without access to crucial preventative services and drastically increased the risk for an outbreak of HIV or HCV in the region. The lack of syringe access also disenfranchised many of the county’s low-income and unhoused residents from needed healthcare services, including Hepatitis A vaccinations, HIV/HCV testing & counseling services, and referrals to wound care and medication-assisted treatment (MAT). Given that SSPs often represent its participants’ sole interface with a healthcare provider [21], we consider these developments unacceptable.

## Reestablishment of services

To address the vital necessity for harm reduction services in Orange County, the Harm Reduction Institute (HRI) was established in August 2019 by medical students at the University of California, Irvine (UCI). HRI operates at the American Addiction Institute of Mind and Medicine (AAIMM), a small intensive outpatient clinic in Santa Ana. From August 2019 to June 2020, HRI distributed naloxone on the streets of Santa Ana, Anaheim, Orange, and Placentia. After securing funding from the CDPH Office of AIDS, HRI launched a weekly physician-authorized harm reduction program in June 2020 offering syringe access and disposal under the medical direction of Dr. Banimahd. Given the legal action taken by the county against OCNEP, and the ease with which Orange County municipalities have passed anti-SSP ordinances, physician-authorized syringe access appears to be the only legally-sound way to operate an SSP in this region.

The authors acknowledge the challenges of operating a harm reduction program at a medical clinic, particularly one which offers MAT and other treatment options for substance use disorder. Physicians and other members of the medical community shoulder much of the blame for the acceleration of the opioid crisis, and they have failed in many instances to provide compassionate and non-judgmental care to PWUDs. As a result, a lingering mistrust of medical professionals exists in the PWUD community. Although the HRI physician-authorized model may appear inconsistent with harm reduction orthodoxy, it currently represents the most viable option for sustained provision of syringe access and other harm reduction services in Orange County. OCNEP adhered closely to the principles established by the broader harm reduction community – but this model unfortunately left OCNEP vulnerable to litigation and harassment. Although OCNEP was ultimately forced to close, it laid the foundation for harm reduction in Orange County and demonstrated the necessity of retaining a physician to supervise harm reduction services in hostile jurisdictions. Operating at a physician’s office has actually facilitated the inclusion of individuals with lived experience in HRI operations, and to our knowledge, our participants have never taken issue with our location in a medical clinic. Most importantly, HRI fosters a culture of respect for participant autonomy. The decision to enter MAT ultimately rests with each individual participant.

Since launching in August 2019, HRI has distributed more than 12,500 doses of naloxone and has tallied 1258 reported overdose reversals. HRI served nearly 900 individual participants in the first 6 months of its on-site program. Members of the community once again have reliable access to sterile syringes, safe syringe disposal, safer injection supplies, and naloxone. HRI also provide our participants with referrals to other community-based organizations such as shelters, transitional housing programs, and food pantries. In spite of its successes, HRI has encountered predictable opposition from municipal officials since commencing operations. The Santa Ana City Council passed legislation in October 2020 to ban state-authorized SSPs from operating in Santa Ana. This ordinance mirrors existing laws codified by other Orange County municipalities, although it notably does not apply to physician-authorized syringe access programs. The City of Santa Ana subsequently resorted to issuing our program baseless zoning citations – however, these citations have had a similarly negligible effect on our ability to serve our participants.

## Conclusion

HRI has been built by the hard work of young people who recognize a moral imperative to address the healthcare needs of people who have no other recourse within the healthcare establishment. We embrace the advancement of individual and population health as our life’s purpose, and we hold a strong conviction that harm reduction services are absolutely essential for many members of our community.

The risks posed by continued attacks on syringe access in Orange County are monumental. In the face of ongoing opposition, the HRI team will continue to maintain a service which will meet the needs of our participants with sustained longevity. For the individuals we serve, access to sterile syringes and other healthcare services constitute a matter of life and death. We hope this inherent urgency will promote action and solidarity not simply among our peers at other institutions, but also among established physicians in Orange County and elsewhere who presently have the power to support and implement the evidence-based intervention of syringe access. The vitality of this work cannot be understated. In pursuing these ends, we will collectively lay the groundwork to allow our present and future patients to achieve the health and wellness deserved by all human beings.

## Data Availability

Not applicable.

## Abbreviations

AAIMM: American Addiction Institute of Mind and Medicine
CDPH: California Department of Public Health
HCV: Hepatitis C Virus
HIV: Human Immunodeficiency Virus
HRI: Harm Reduction Institute
MAT: Medically-Assisted Treatment
OCHCA: Orange County Health Care Agency
OCNEP: Orange County Needle Exchange Program
PLWH: Person(s) living with HIV
PWUD: Person(s) who use drugs
SSP: Syringe services program
UCI: University of California, Irvine
